# A novel method for detecting credit card fraud problems

**DOI:** 10.1371/journal.pone.0294537

**Published:** 2024-03-06

**Authors:** HaiChao Du, Li Lv, Hongliang Wang, An Guo

**Affiliations:** 1 Shenyang Institute of Computing Technology, Chinese Academy of Sciences, Shenyang, China; 2 University of Chinese Academy of Sciences, Beijing, China; 3 Liaoning Province Digital Twin-based Interactive System Engineering Research Center, China; 4 School of Computer & Information Engineering, Anyang Normal University, Anyang, Henan Province, China; 5 Key Laboratory of Oracle Bone Inscriptions Information Processing, Ministry of Education of China, Anyang, Henan Province, China; St Xavier’s Catholic College of Engineering, INDIA

## Abstract

Credit card fraud is a significant problem that costs billions of dollars annually. Detecting fraudulent transactions is challenging due to the imbalance in class distribution, where the majority of transactions are legitimate. While pre-processing techniques such as oversampling of minority classes are commonly used to address this issue, they often generate unrealistic or overgeneralized samples. This paper proposes a method called autoencoder with probabilistic xgboost based on SMOTE and CGAN(AE-XGB-SMOTE-CGAN) for detecting credit card frauds.AE-XGB-SMOTE-CGAN is a novel method proposed for credit card fraud detection problems. The credit card fraud dataset comes from a real dataset anonymized by a bank and is highly imbalanced, with normal data far greater than fraud data. Autoencoder (AE) is used to extract relevant features from the dataset, enhancing the ability of feature representation learning, and are then fed into xgboost for classification according to the threshold. Additionally, in this study, we propose a novel approach that hybridizes Generative Adversarial Network (GAN) and Synthetic Minority Over-Sampling Technique (SMOTE) to tackle class imbalance problems. Our two-phase oversampling approach involves knowledge transfer and leverages the synergies of SMOTE and GAN. Specifically, GAN transforms the unrealistic or overgeneralized samples generated by SMOTE into realistic data distributions where there is not enough minority class data available for GAN to process effectively on its own. SMOTE is used to address class imbalance issues and CGAN is used to generate new, realistic data to supplement the original dataset. The AE-XGB-SMOTE-CGAN algorithm is also compared to other commonly used machine learning algorithms, such as KNN and Light GBM, and shows an overall improvement of 2% in terms of the ACC index compared to these algorithms. The AE-XGB-SMOTE-CGAN algorithm also outperforms KNN in terms of the MCC index by 30% when the threshold is set to 0.35. This indicates that the AE-XGB-SMOTE-CGAN algorithm has higher accuracy, true positive rate, true negative rate, and Matthew’s correlation coefficient, making it a promising method for detecting credit card fraud.

## 1. Introduction

Credit cards, born with the development of electronic banking, are widely used all over the world, for example, in the US alone there are about 100 billion credit cards currently in use [1]. However, credit card fraud is a major problem faced by banks and credit card companies worldwide, resulting in huge revenue losses and posing a threat to the financial health of institutions. Therefore, anomaly detection technology has become an important means to prevent credit card fraud. This study focuses on the improvement of smote, gan, autoencoder, xgboost to detect credit card fraud. Before introducing this method, we first provide a detailed explanation of the research status on credit card fraud detection. In past studies, credit card fraud detection has been a hot research topic. Traditional methods of fraud detection have been effective to a certain degree, but they have significant limitations and shortcomings. Let’s analyze some of these methods and their drawbacks before introducing a new approach: AE-XGB-SMOTE-CGAN.

The traditional fraud detection methods include Rule-Based Systems, Statistical Methods, and Machine Learning (ML) Methods.

Rule-Based Systems: These are systems built on predefined rules to identify fraudulent transactions. They often incorporate business intelligence and expert inputs.

Limitations: They can miss new fraud patterns and generate a high rate of false positives. The system is rigid and requires continuous manual effort to update the rules as fraud patterns evolve.

Statistical Methods: Commonly used methods like mean, median, standard deviation, and clustering algorithms.

Limitations: These methods work well with normal distributions but struggle with non-normal distributions. They also tend to perform poorly with high-dimensionality data.

Machine Learning (ML) Methods: Algorithms such as Decision Trees, Logistic Regression, and Support Vector Machines are used to predict fraudulent cases.

Limitations: ML methods need a large amount of labeled data to train the models. In fraud detection, there’s a class imbalance problem where the number of fraud cases is significantly less than non-fraud cases. This can result in models that are biased towards non-fraud cases.

Another main challenge is the imbalance of data, that is, fraudulent transactions are rare compared to legitimate transactions, which may result in biased detection models. Oversampling is a commonly used pre-processing technique, as under sampling may remove important information and result in inaccurate classification. However, oversampling also has its limitations, as it can include illegitimate samples, which is still an active area of research. Among various oversampling methods, Synthetic Minority Over-sampling Technique (SMOTE) is considered a "de facto" standard due to its simplicity and effectiveness. However, SMOTE may not always generate diverse samples, which can be a potential drawback.

In the field of deep learning, researchers have used different architectures such as convolutional neural networks, recurrent neural networks, and generative adversarial networks (GAN) to solve the problem of fraud detection. Generative Adversarial Networks (GANs) and their variants are frequently employed for generating new synthetic samples. Although initially designed for generating realistic images for large datasets, GANs can also generate samples from the minority class, thereby balancing the class distribution and effectively preventing overfitting. However, GAN is not a perfect fit for oversampling, as it was originally designed for generating realistic-looking images using convolutional neural networks (CNNs), rather than producing over-samples for the minority class. Furthermore, GAN may encounter a data scarcity problem, as the minority class is already in a reduced form where model training requires sacrificing more of its data for validation and testing purposes. SMOTE-CGAN is a new deep learning technology that can achieve promising results in different datasets, including credit card transactions. It combines SMOTE technology and CGAN generative model to generate synthetic minority samples for fraudulent transactions, thereby improving the performance of the model. This paper proposes a new credit card fraud detection algorithm, which integrates and improves four algorithms: SMOTE, GAN, AutoEncoder, and XGBoost. This algorithm is referred to as AE-XGB-SMOTE-CGAN.

The AE-XGB-SMOTE-CGAN algorithm, which combines machine learning and deep learning, can achieve accurate and efficient prediction of fraudulent data even in imbalanced data scenarios. The overall structure of the AE-XGB-SMOTE-CGAN algorithm consists of an autoencoder, a xgboost, a smote and a cgan. Firstly, SMOTE generates promising samples which is then “transferred” to GAN which no longer uses random sample [2]. And then the autoencoder is used to train the generated data and extract the feature data, the feature data is fed into the xgboost algorithm for classification prediction to determine if the credit card transaction data is normal or abnormal. Unlike other methods that adopt a binary (0/1) classification for the XGBoost, the AE-XGB-SMOTE-CGAN adopts xgboost with probabilistic classification, which further improves its performance. Detailed information will be described in the following chapters. In summary, using AE-XGB-SMOTE-CGAN to detect credit card fraud is one of the current hotspots of research.

To validate the idea behind our algorithm, we used a dataset of credit card transactions from a banking institution.

This dataset represents transactions that took place within a period of two days. Among the 284,807 transactions, there were 492 cases of fraud. This dataset is highly imbalanced, with the positive class (frauds) accounting for only 0.172% of all transactions. The input variables are all numerical, and were generated through a PCA transformation. Unfortunately, the original features and other background information about the dataset cannot be provided due to confidentiality reasons. The principal components obtained from the PCA are represented by features V1-V28. The only two features that were not transformed by PCA are ’Time’, which indicates the time elapsed between transactions, and ’Amount’, which indicates the transaction amount. The ’Class’ feature is the response variable, and has a value of 1 for cases of fraud and 0 for legitimate transactions. The ’Amount’ feature can be used for cost-sensitive learning.

AE-XGB-SMOTE-CGAN uses smote generates promising samples and then the samples “transferred” to GAN which no longer uses random sample. AE-XGB-SMOTE-CGAN、xgboost、smote、cganAE-XGB-SMOTE-CGANxgboost AE-XGB-SMOTE-CGAN The proposed model was assessed based on MCC, TPR, TNR, and ACC metrics, and the experimental findings revealed its superior performance compared to established models like KNN, XGBoost, and Random Forest.

In the following sections, we will provide an overview of the research status on credit card fraud detection in both domestic and international contexts. Specifically, Section 3 will explain the AE-XGB-SMOTE-CGAN algorithm we used, including the network structure and algorithmic flow. Additionally, Section 4 will detail the experiments we performed, and Section 5 will conclude our research.

## 2. Related work

The rapid development of electronic payments has made credit card payment the preferred choice for young people when shopping. Its advantage of pre-payment and installment consumption solves the problem of young people not being able to purchase the products they like due to temporary shortage of funds. However, some unscrupulous users attempt to misuse the normal behavior of paying by credit card and maliciously overdraft the credit card. These people are often difficult to repay the credit card, which poses a great financial risk to banks and other financial institutions. To address this issue, many researchers in the industry have tried using machine learning and deep learning algorithms to detect credit card fraud.

Research on anomaly detection of bank credit card fraud based on SMOTE-GAN is active both domestically and internationally, and researchers have developed several effective models to deal with the issue. Maram Alamri and Mourad Ykhlef proposed a credit card fraud detection method based on sampling techniques [3]. Their method involves the use of the SMOTE algorithm to ensure a balanced representation of positive and negative samples in the training dataset. In this article, they provide an overview of various sampling techniques and their different methodologies. They also examine how imbalanced data affects the performance of learning algorithms, leading to inaccuracies, incorrect results, and reduced F1 evaluation, recall, and precision scores. Finally, they discuss the significance of sampling techniques in addressing the challenge of imbalanced data in credit card fraud detection.

However there are limitations to under sampling and oversampling when applied alone that lead to a decreased performance of the detection system. When applying under sampling, these include the removal of important information and confusion between information data and noise, which can negatively affect the overall classification performance while oversampling can lead to overfitting and overlapping.

The optimal solution is to apply a hybrid of the best sampling techniques in order to balance the dataset and improve the performance of the detection system.

Recently, Choi et al. [4] applied the Conditional GAN framework to the credit card dataset to generate synthetic data. Conditional GANs generate a synthetic credit card dataset, which can be used indistinguishably for training without revealing the actual dataset. The findings of their study show that deep learning techniques can be used with excellent outcomes to generate synthetic credit card data. However, despite the enormous progress made in GAN techniques, these models still have shortcomings in dealing with credit card fraud. Therefore, future investigation should prioritize the development of advanced and effective GANs to enhance the existing financial models.

In another study, Lee and Park [5] argued that deep learning methods perform better than machine learning techniques when dealing with large quantities of datasets, such as credit card transaction datasets. The authors argue that previous research on addressing the challenge of imbalanced classes has limitations stemming from overfitting and loss of data. To overcome these issues, they utilize GAN and their proposed model for handling imbalanced classes. They then evaluate the detection ability of their model using Random Forest classification, with data augmentation based on GAN. Results indicate that their proposed model outperforms models that do not account for imbalanced classes, and is superior to previous approaches.

[6] some researches proposed some semi-supervised methods [23] based on autoencoders in anomaly detections [7, 8]. They trained deep autoencoders on the data samples with no anomalies and detected anomalous events according to the reconstruction errors of anomalous and normal samples. The autoencoders can learn better representations of samples to improve classification effectiveness.

In reference [31] a novel unsupervised clustering method based on an autoencoder (AE) is proposed for credit card fraud detection. The method utilizes an AE architecture with three hidden layers in both the encoder and decoder components. Activation functions such as exponential linear unit (ELU) and rectified linear unit (ReLU) are employed in different layers of the AE. The optimizer used in the experiments is root mean square propagation (RMSProp), which yields the best results.

The AE-based clustering method demonstrates effective classification performance, as illustrated in reference [31]. By appropriately selecting a threshold for AE reconstruction errors, the method successfully separates fraudulent data from normal data. This approach offers a promising solution for credit card fraud detection without requiring labeled training data.

Overall, researchers achieved good results in applying their proposed model to bank credit card fraud anomaly detection, but challenges remain, such as ensuring continued high accuracy of the model at different time periods and dealing with large-scale datasets.

In light of the above, this paper proposes a novel method for credit card fraud detection problems, we can call it AE-XGB-SMOTE-CGAN method, and through the training of this model, our algorithm shows better performance on the above datasets.

## 3. The proposed method

In this paper, we propose a novel hybrid approach that combines the strengths and overcomes the deficiency of two independent models that include SMOTE and GAN. SMOTE is known to produce some irregular or “out of distribution” samples. Additionally, SMOTE has not been generally used with deep learning and GAN generally has not been used for small datasets (minority classes)

As just mentioned, the paper proposes a combining autoencoder, xgboost, smote, gan algorithm, we call it AE-XGB-SMOTE-CGAN method. The proposed AE-XGB-SMOTE-CGAN method uses the AutoEncoder to extract data features and employs the xgboost with probabilistic classification to classify credit card transactions as normal or fraudulent. To describe the AE-XGB-SMOTE-CGAN method clearly, xgboost.

### 3.1 Autoencoder

**Fig 1:**
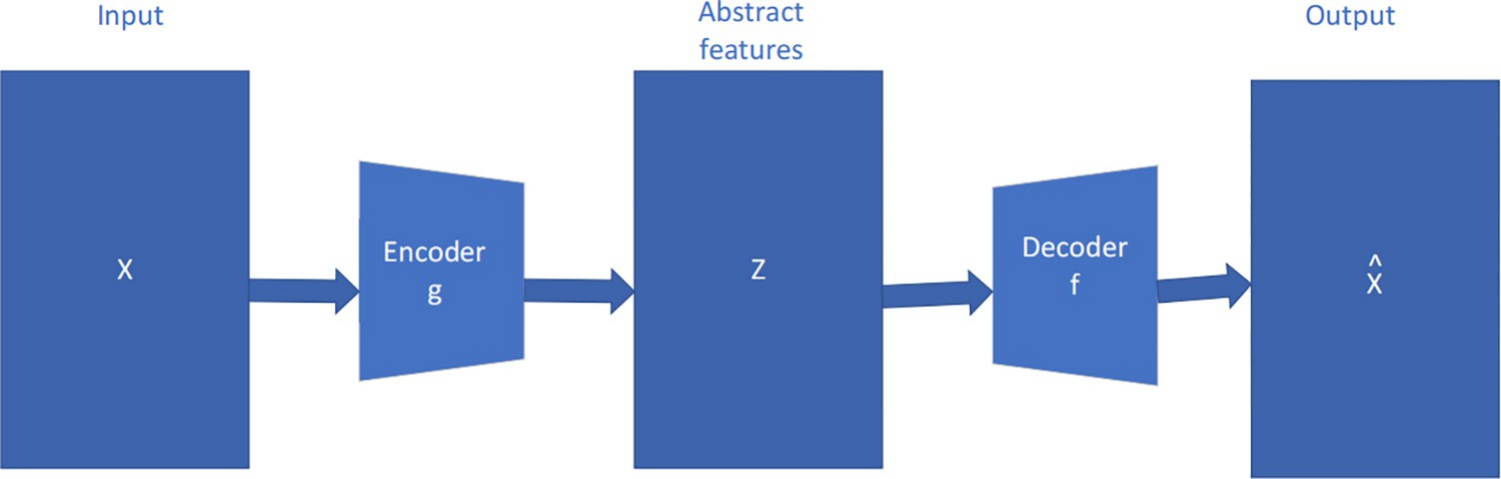
Typical autoencoder structure.

The autoencoder algorithm is a widely used unsupervised learning method for reducing the dimensionality of data. It is based on neural networks and aims to convert high-dimensional data into lower-dimensional data without supervision. This is similar to PCA, but offers more flexibility as it is not limited by linearity. The autoencoder [9] model typically consists of two symmetrical parts: the encoder and decoder. The encoder compresses the original high-dimensional input data, while the decoder reconstructs the original data in its original high-dimensional form. The overall structure is shown in the following Fig 1.

The left part is the Encoder layer. The Encoder layer is a crucial component of the autoencoder architecture, which is comprised of a sequence of layers with nodes that encode the input into the subsequent layer. Conversely, the right part of the architecture is the Decoder layer, which is the reverse image of the Encoder architecture with the same number of nodes as the input. The input sample x is mapped to a feature space z through an encoder (g), which is the encoding process. The abstract features in z are then mapped back to the original space to obtain a reconstructed sample x’ through a decoder (f), which is the decoding process. The optimization objective is to minimize the reconstruction error, thereby simultaneously optimizing the encoder and decoder, and learning an abstract feature representation z tailored to the input sample x. The encoding process of the original data X from the input layer to the hidden layer is the following Eq (1):

z=gθ1(x)=σ(w1x+b1) (1)

$$\hat{x}=f\theta 2(h)=\sigma (w2z+b2)$$ (2)

The decoding process of the original data X from the hidden layer to the output layer is the following Eq (2):

The difference between the input and the output is called the reconstruction error. The AutoEncoder model is trained with the goal of minimizing the reconstruction errors.

### 3.2 XGBoost

XGBoost (short for "Extreme Gradient Boosting") is a popular machine learning algorithm known for its high predictive accuracy and computational efficiency. It is a decision-tree-based ensemble learning method that combines multiple weak classifiers to create a strong classifier. XGBoost has gained widespread popularity in various fields, including finance, healthcare, and natural language processing [10].

**Fig 2:**
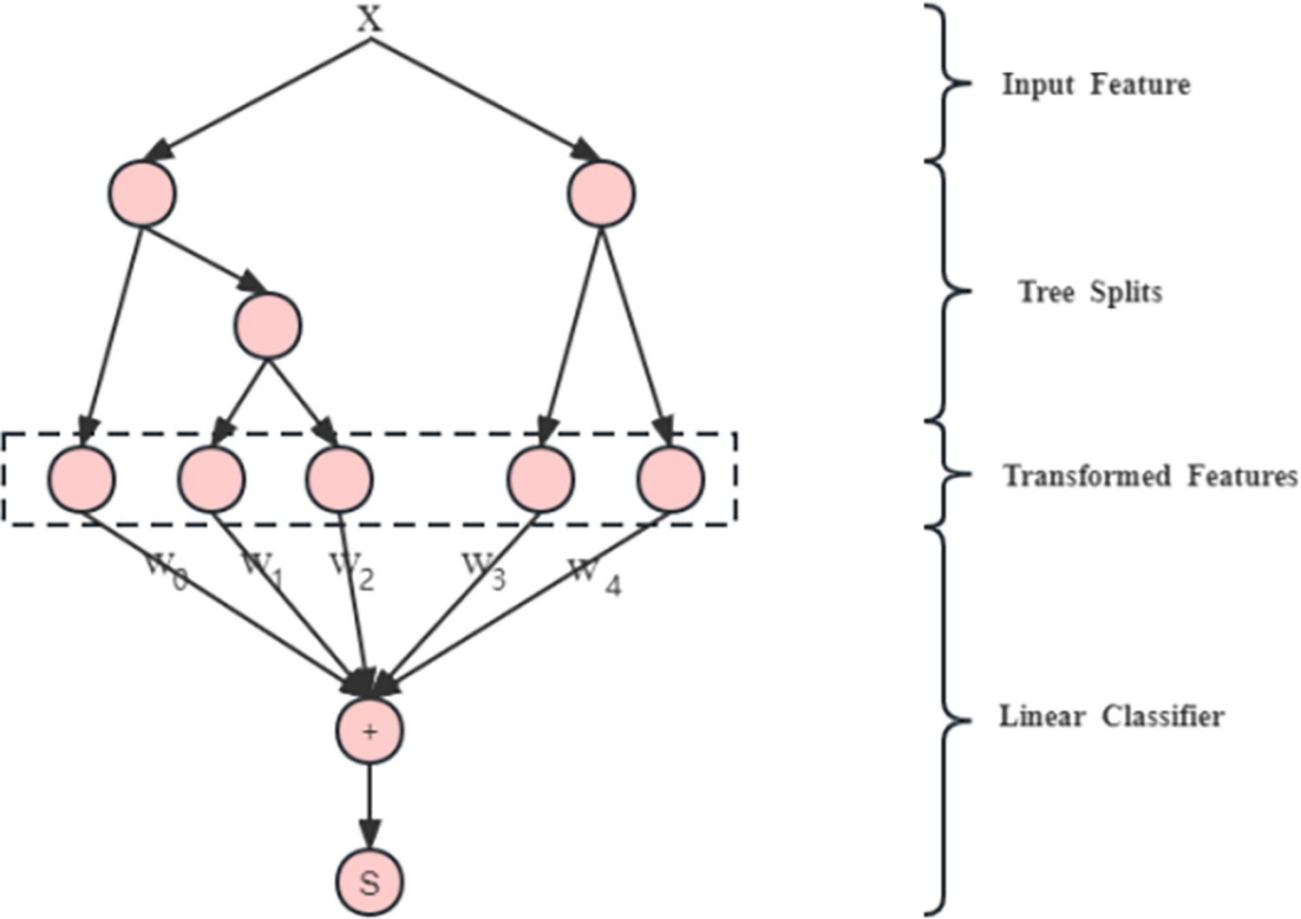
XGBoost tree structure.

The algorithm works by iteratively adding decision trees to a model and adjusting the weights of misclassified samples. XGBoost also incorporates regularization techniques, such as L1 and L2 regularization, to prevent overfitting and improve generalization performance. Additionally, it uses a distributed computing system to parallelize the training process, making it scalable and efficient for large datasets [11]. The XGBoost tree structure is shown in Fig 2.

In addition to the standard decision tree structure, XGBoost also includes some advanced features such as handling missing values, weighted quantile sketch for approximate splitting, and parallel computation to speed up the training process.

Overall, XGBoost’s tree structure enables the algorithm to learn complex nonlinear relationships between features and the target variable, leading to highly accurate predictions.

### 3.3 Synthetic Minority Oversampling Technique (SMOTE)

SMOTE is an effective algorithm for addressing imbalanced class problems that involves over-sampling of the minority class and under-sampling of the majority class [12]. This approach utilizes k-nearest neighbors to randomly select neighboring points for interpolation, with a threshold between 0 and 1. Unlike traditional sampling methods, SMOTE samples in feature space rather than data space, resulting in more accurate outcomes. Various variants of SMOTE have been successfully used in diverse application domains, such as bioinformatics, fault detection, high-dimensional gene expression datasets, and video surveillance. Examples of SMOTE variants include Borderline-SMOTE, SVM-SMOTE, KMeans-SMOTE, and regular SMOTE. Numerous SMOTE variants have been implemented in Python libraries.

Pseudocode for this algorithm is provided in Algorithm 1. The parameters n and d represent the size and dimension of the minority class, respectively, while N is the size of the majority class, and k is the parameter for the k-nearest neighbor. Lines 1–5 identify the KNN for each minority sample and then use them for interpolation to create new samples. Lines 6–12 describe the interpolation step, where N-n samples are created and added to the minority class. It is not a completely random sampling method, but rather uses interpolation between neighboring minority class examples to generate new samples. It is efficient and easy to implement. Each minority example receives k nearest neighbors (KNN), which are randomly selected for interpolation to create new samples.

**Algorithm 1:** SMOTE Algorithm

**Input:** d-dimensional minority samples X of size n from a training data set of size N that requires N-n over-samples. The parameters n and d are the size and dimension of the minority class respectively; N is the size of the majority class and parameter k for k -nearest neighbor

**Output:X**

1: N ← N-n

2: for i = 1:||X||do

3:   S←KNN(xi,k)//xi∈X

4:   X ← interpolate(N/100,xi,S)//for N > 100

5: end

6: interpolate(N,xi,S)

7: While ||X||< N do

8: $a\leftarrow R_{1}^{k\times 1}(1)$//pick a randow value from 1..k

9: *X*_*j*_←*S*{*a*}

10: //$\Delta x_{ij}=x_{j}-x_{i}\Rightarrow euclidean\;distance\;between\;x_{j}\;and\;x_{i}$

11: //$R_{D}^{1\times d}\Rightarrow a\;decimal\;random\;number\;between\;0\;to\;1$

12: $X\leftarrow X\cup (x_{i}+\Delta x_{ij}\times R_{D}^{1\times d})$

13: end

14: return X

15: 

However, a major limitation of SMOTE is that it tends to focus on local information, which results in the generation of a less diverse set of data. The data generated by SMOTE are often less realistic than those produced by GAN, which captures the true data distribution to generate data for the minority class.

### 3.4 Generative Adversarial Network (GAN)

GAN is a broader machine learning framework that uses two networks in a feedback loop to generate new data that resembles a given dataset [13]. GAN was initially designed for generating realistic images, but its success has led to the development of various derivative models, including CGAN. These models have been instrumental in solving the problem of unbalanced datasets and are likely to become increasingly important in the future [14].

The GAN architecture comprises two networks, as previously mentioned, where the generator network’s aim is to create data that deceives the discriminator network into identifying it as "real." [15]. To optimize its performance, the discriminator loss should be maximized when data comes from the generator. The generator’s objective is to generate data that the discriminator can identify as "real." To optimize the discriminator’s performance, the loss when supplied with batches of both genuine and generated data should be minimized.The objective of the discriminator is to not be “fooled” by the generator. The following is the engineering formula of GAN network;

$$\min_{G}\max_{D}V(D,G)=\max E_{X\;pdata(x)}[\log D(x)]+E_{Zz(z)}[\log(1-D(G(z)))]$$ (3)

The equation represents a generator that creates a distribution based on a noise distribution p(z), and learns the distribution of real data x. The generator G is used to generate data, while the discriminator D is used to distinguish between generator and real data distributions. The learning process is complete when the discriminator cannot distinguish the source of the data, meaning it believes there is a 50% chance the data comes from real data distribution and a 50% chance it comes from the generator. The generated data is random and unpredictable, and there is weak control over the target generation.

However The original GAN has many drawbacks. The generator produces a limited range of outputs that do not cover the entire data distribution [16]. This can lead to poor generalization and a lack of diversity in the generated samples. Another limitation is the generated data is random and unpredictable, and it is not possible to control the network to output specific data. The generation target is not clear, and the controllability is weak. To address these issues, CGAN has been proposed. CGAN stands for conditional generative adversarial network [17], which is an advanced type of neural network that can be used to generate new data based on specified conditions. This technique has been particularly useful for addressing the challenges posed by unbalanced datasets [18], which are commonly encountered in various fields. The following is the engineering formula of CGAN network [19].


$$\min_{G}\max_{D}V(D,G)=\max E_{X\;pdata(x)}[\log D(x|y)]+E_{Zz(z)}[\log(1-D(G(z|y)))]$$ (4)

The equation for a Conditional Generative Adversarial Network (CGAN) includes an additional condition y, which is used to impose constraints on both the generator and discriminator based on any labels included in the training data. CGAN differ from regular GAN as both the generator and discriminator include this additional condition, which can assist in guiding the data generation process and producing outputs that better align with desired outcomes. As a result, the generated data is unpredictable and random, with limited control over the specific features of the output [20].

By producing synthetic data that adheres to specific conditions, CGAN can help reduce bias and improve predictions in data analysis.

**Fig 3:**
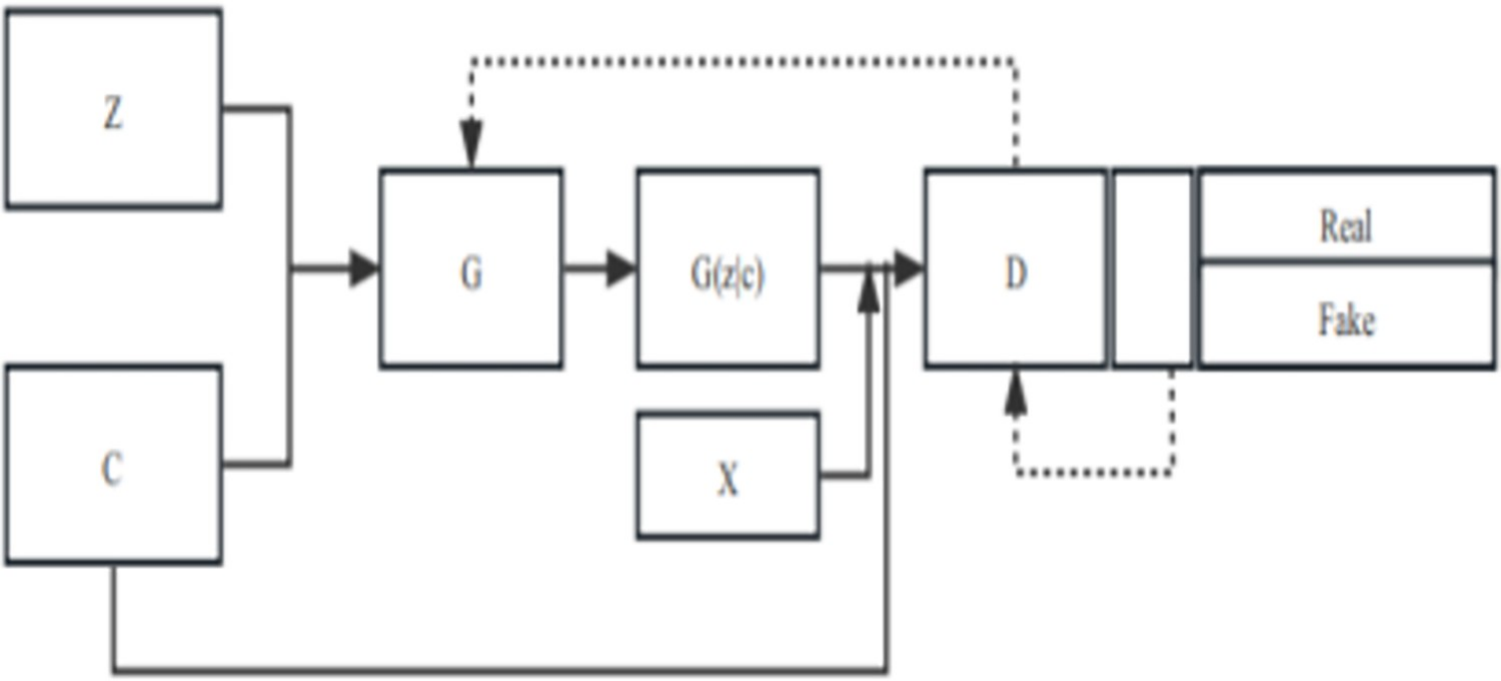
The structure of CGAN.

One such application of CGAN technology is in demographic data synthesis, where generating representative samples of under-represented groups can be challenging. Another application is in medical data processing, where accurate predictions depend on having a diverse and balanced dataset [21]. Financial fraud detection is another area where CGAN have been used to generate synthetic data that helps uncover patterns and trends in large datasets [22].The structure diagram for CGAN is shown in Fig 3.

CGAN consists of two neural networks, a generator network and a discriminator network. The generator network generates synthetic data based on specific conditions provided by the user, while the discriminator network evaluates the authenticity of the generated data by comparing it to the real data. The two networks then provide feedback to each other in a process called adversarial training, where they learn and improve their performance over time. The result is a generator network that can produce new data that closely resembles the real data and adheres to specific conditions set by the user [23].

**Algorithm 2:** CGAN Algorithm

**Input: The training dataset examples x and noise samples z are generated from an appropriate random number generator. An optional parameter can be the size of the fake example sample needed, denoted by n fake.**

**Output:u**

1: //initialize parameters

2: //m_i is minibatch indices for *i*^*th*^ index and T is total iterations

3: CGAN(x,z,y,n fake)

4: for t = 1:T do

5: //generally step size S is 1

6: //subscript d and g refers to discriminator and generator entity //respectively

7:  for s = 1:S do

8:   $g_{d}\leftarrow SGD(-logD(x|y)-log(1-D(G(z|y)),w_{d},m_{i}))$

9:   $w_{d}\leftarrow weights(g_{d},w_{d})$

10:  end

11: $g_{g}\leftarrow SGD(-logD(G(z|y)),w_{g},m_{i}))$

12: $w_{g}\leftarrow weights(g_{g},w_{g})$

13: end 

14: $u\leftarrow collectFakeEx(Model_{d}(W_{d},x,z),Model_{g}(W_{g},x,z),n_{fake})$

15: return u

16: 

However, CGAN is not the most suitable option for oversampling, as it was originally developed to generate realistic images using convolutional neural networks (CNNs), rather than producing oversamples for minority classes. Moreover, CGAN may encounter data scarcity issues, because the minority class is already in a reduced form, and model training requires sacrificing more of its data for validation and testing purposes. Nevertheless, cross-validation techniques may help mitigate this problem to some extent.

### 3.5 The AE-XGB-SMOTE-CGAN method

In this paper, we propose an AE-XGB-SMOTE-CGAN method, in which we use transfer learning to address the limitations of SMOTE and CGAN mentioned in the earlier part of the paper when performing data augmentation. Specifically, our approach extracts knowledge about the minority class from SMOTE and applies it to CGAN. This approach seeks to diversify the original samples generated by SMOTE through CGAN, while also improving their quality by emulating them with realistic samples.

**Fig 4:**
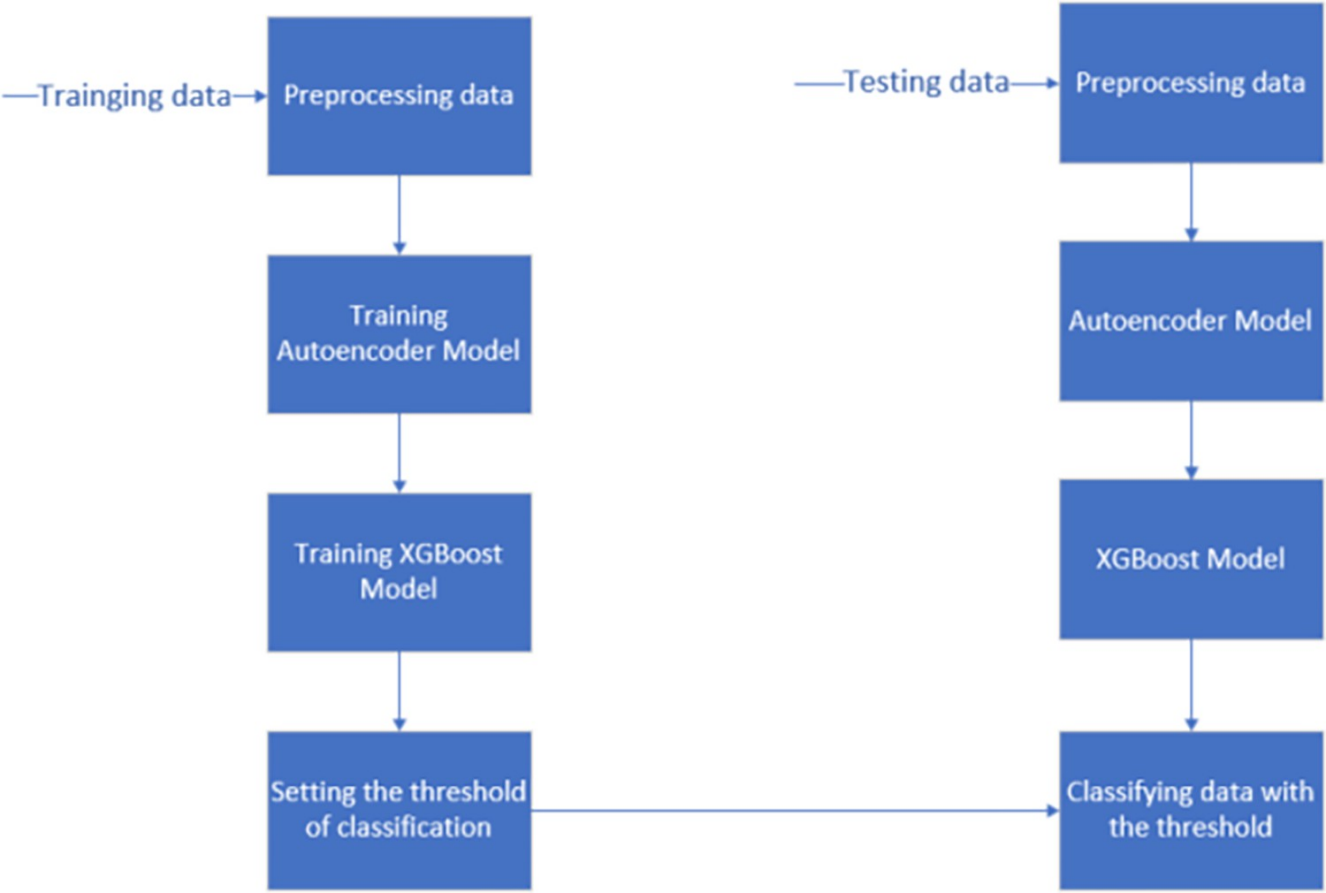
The flowchart of AE-XGB-SMOTE-CGAN method.

**Fig 5:**
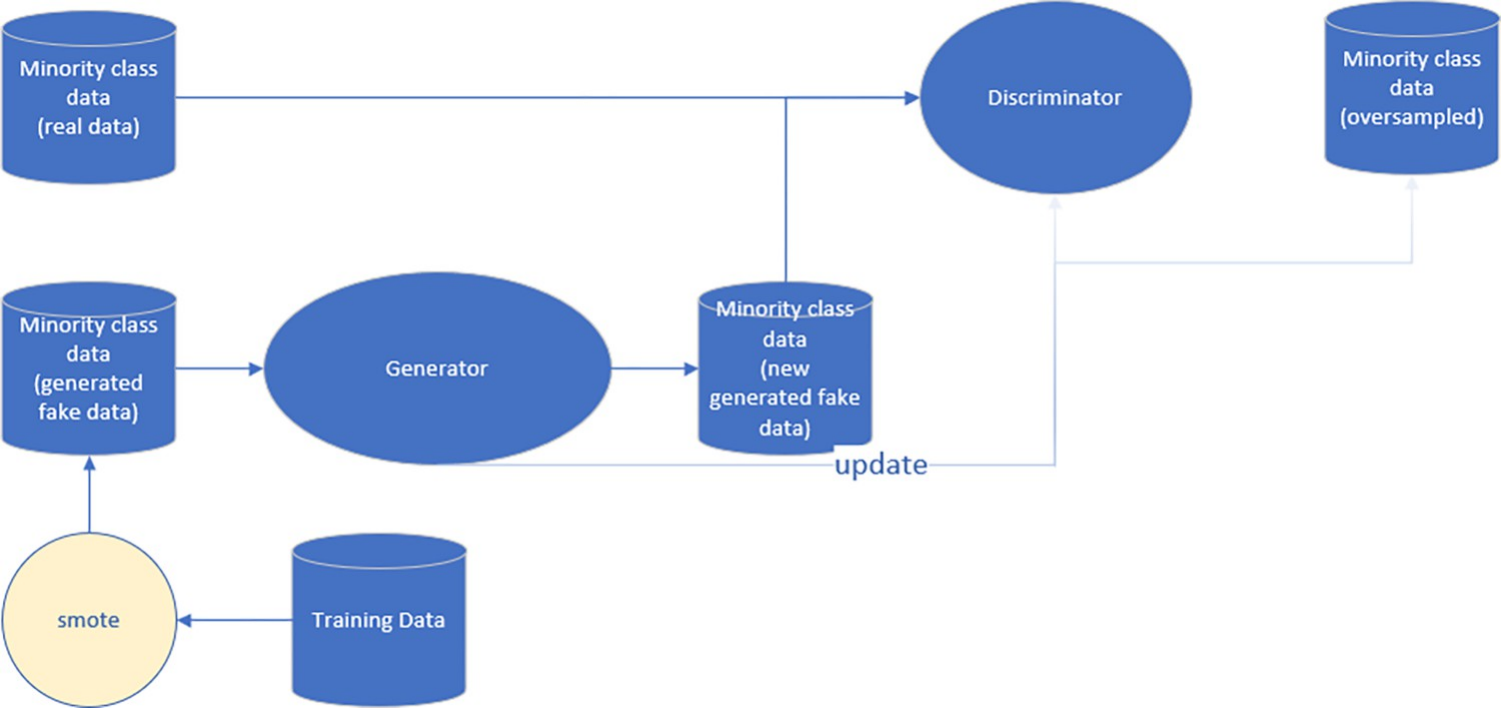
The Process of sample generation with SMOTE-CGAN.

Fig 4 is the flowchart of the AE-XGB-SMOTE-CGAN method. First, the data is pre-processed, abnormal data is processed, data normalization is performed. To improve the performance of model, data augmentation through SMOTE and CGAN algorithms is a powerful technique. During the stage of data preprocessing, the cleaned data is subjected to data augmentation using SMOTE and CGAN techniques. Fig 5 is the process of sample generation with SMOTE-CGAN, which combines the strengths of SMOTE and GAN while overcoming their weaknesses. SMOTE-CGAN uses transfer learning to extract knowledge about the minority class from SMOTE and applies it to CGAN in order to diversify the original samples. Furthermore, we improved the sample quality by emulating realistic samples. Overall, our approach provides a unique way to augment data in order to achieve better results.

The formalization of CGAN in the AE-XGB-SMOTE-CGAN method is not significantly different from that of the original GAN. The main modification involves replacing the random generator function of GAN with a repertoire of oversampled minority examples from SMOTE. The modified scores can be expressed as follows:

discriminator score:

$$\max V(D)=\max_{D}E_{x^{*}p_{data}(x^{*})}[\log D(x^{*}|y)]+E_{u}[\log(1-D(G(u|y)))]$$ (5)

Generator score:

$$min(G)=min_{D}-E_{u}[\log(D(G(u|y)))]$$ (6)

Here, x* represents the training samples of the minority class(es), while u denotes the oversampled data of the same class(es) generated using various algorithms, such as SMOTE in this particular case.

After the data is separated into training, validation, and test sets, we proceeded with testing using the original data with data augmentation. The training set was used to train the autoencoder framework, where the weights and parameters were adjusted through backpropagation to obtain an autoencoder model. The autoencoder model’s feature coding for dimensionality reduction was then fed into the xgboost network for further training. A certain output probability was utilized to determine if the data was fraudulent or normal. It was important to identify the appropriate classification probability threshold to ensure optimal performance during training the xgboost model. To verify the effectiveness of the models, the autoencoder and xgboost models, along with the determined threshold value, were applied to each test data point. This allowed us to categorize each test data point as fraudulent or normal based on the trained models and threshold.

In the AE-XGB-SMOTE-CGAN method, the xgboost model with probabilistic classifications is used to classify a datum as fraudulent with probability p and as normal with probability 1 − p, where 0 ≤ p ≤ 1. Subsequently, AE-XGB-SMOTE-CGAN outputs the final classification as fraudulent if p is greater than a predetermined classification probability threshold θ. Therefore, fine-tuning the probability threshold value θ can provide a customized classification result for AE-XGB-SMOTE-CGAN.

The pseudocode of the proposed AE-XGB-SMOTE-CGAN method is shown as Algorithm 1 below.

**Algorithm 3:** AE-XGB-SMOTE-CGAN Algorithm

**Input:** Xtrain: trainging data; Xvalidation: validation data; Xtest: test data;

**Output:** the classification result for every test data, the result is 0 or 1,0 represents normal and 1 represents fraudulent;

16: // User-defined parameter $k$ for $k$ -nearest neighbors.

17: // First execute SMOTE given in Algorithm 1 then CGAN given in Algorithm 2

18: u← = call Algorithm_1 (x*,k) // generate over-sampled minority examples $u$.

19: Xtrain← = call Algorithm_2 (x*,u,y,N−n).

20: Train the autoencoder model AEMT with Xtrain

21: MT ← AEMT(Xtrain)

22: Train the xgboost model XGT with MT

23: MV ← AEMT(Xvalidation)

24: for ϴ ← 0 to 1 step 0.01

25:  for each v in MV do

26:  p ← XGT(v)

27:  if p > ϴ then

28:  result[ϴ][v] ← 1

29:  else

30:  result[ϴ][v] ← 0

31:  end if

32:  end for

33: end for

34: Find the best ϴ in terms of metric

35: MC ← AEMT(Xtest)

36: for each c in MC do

37:  q ← XGT(c)

38:  If q > ϴ then

39:   ouput[c] ← 1

40:  end if

41: end for

42: return ouput

The pseudocode is summarized in the following steps:

Step 1:Generate the data u with Algorithm 1, and then input the data u to the Algorithm 2 to generate new data. The generated samples u from algorithm1 is used instead of random noise z of Algorithm 2.

Step 2: The training dataset is then used to train the autoencoder model AEMT, which generates a training dataset with a feature code set M.

Step 3: Fit the xgboost model XGT using the training dataset feature encoding set MT obtained in Step 1.

Step 4: Perform the AEMT process on the validation dataset and obtain the set of feature codes MV from the validation dataset.

Step 5: Ranging from 0 to 1 in increments of 0.01, at each step, perform the following operation: input each codev in MV into the XGT model, gain a probability value p. When p is greater than the threshold value, the result is 1, which is fraudulent data; otherwise, it is 0, which is normal data.

Step 6: Use all the classification results in Step 5 to find the best threshold according to the metric to produce the best classification performance.

Step 7: Apply the AEMT model to the test dataset and extract the test dataset feature code set MC.

Step 8: For each code C in MC, input it into the XGT model to calculate a probability value q. Then, compare q with the optimal threshold determined in Step 6 to derive the classification result.

## 4. Discussion and experimental results

In order to evaluate the effectiveness of our method, we conducted our validation on a credit card transaction dataset. This dataset presents transactions that occurred in two days, where we have 492 frauds out of 284,807 transactions. The dataset is highly unbalanced; the positive class (frauds) accounts for only 0.172% of all transactions.

To address the issue of data imbalance in our modeling process, we first pre-processed the data and removed any anomalies. However, when predicting the model, we may encounter challenges in making accurate predictions due to this imbalance, as the model may tend to predict the majority dataset and fail to account for the minority. To mitigate this problem, we explored different techniques such as under sampling and oversampling. However, we found that under sampling would result in significant data loss, which would negatively impact the model’s training accuracy. On the other hand, oversampling by duplicating minority class samples could increase the complexity of the model and lead to overfitting.

Therefore, we adopted a new technology, which combined both the SMOTE and CGAN algorithms, to enhance the quality of our data. The SMOTE algorithm selects a random sample y from the k-nearest neighbors of each minority class [24] sample x and synthesizes a new sample along the x, y line. This approach can effectively reduce the risk of overfitting in the final model. Moreover, we used CGAN as a means of data enhancement to further improve the quality of our data, which provided a more robust solution for [25] addressing the problem of data imbalance in our modeling process.

### 4.1 Data pre-processing

#### 4.1.1 Datasets

The dataset includes credit card transactions made by cardholders in September 2013. It covers transactions that occurred over a period of two days, with a total of 284,807 transactions, of which 492 were fraudulent.

Before training a model, it is crucial to perform data preprocessing, which involves cleaning the data, selecting the relevant features for model training, and normalizing the features. In this experiment, Table 1 displays a subset of features, including anonymized features labeled v0-v28 with feature values generated through Principal Component Analysis (PCA). We only illustrate six features related to v, where the class feature is the label demonstrating whether a transaction is fraudulent or not. A fraud transaction is labeled as 1, whereas a normal transaction has a label of 0. The Amount feature represents the transaction amount, and since its mean and variance differ significantly from v1-v28, normalization is performed on this feature. The time feature, representing the interval between all transactions and the first transaction, is removed since no time-sequence-related analysis is conducted in this experiment.

**Table 1:** Some features of the dataset from a bank.

Time	V1	V2	V3	V4	V5	V28	Amount	Class
0	−1.359807134	−0.072781173	2.536346738	1.378155224	−0.33832077	−0.021053053	149.62	0
17	0.96249607	0.328461026	-0.171479054	2.109204068	1.129565571	0.129394059	15.99	1
472	−3.043540624	−3.157307121	1.08846278	2.288643618	1.35980513	0.035764225	529	1

#### 4.1.2 Data augmentation

**Fig 6:**
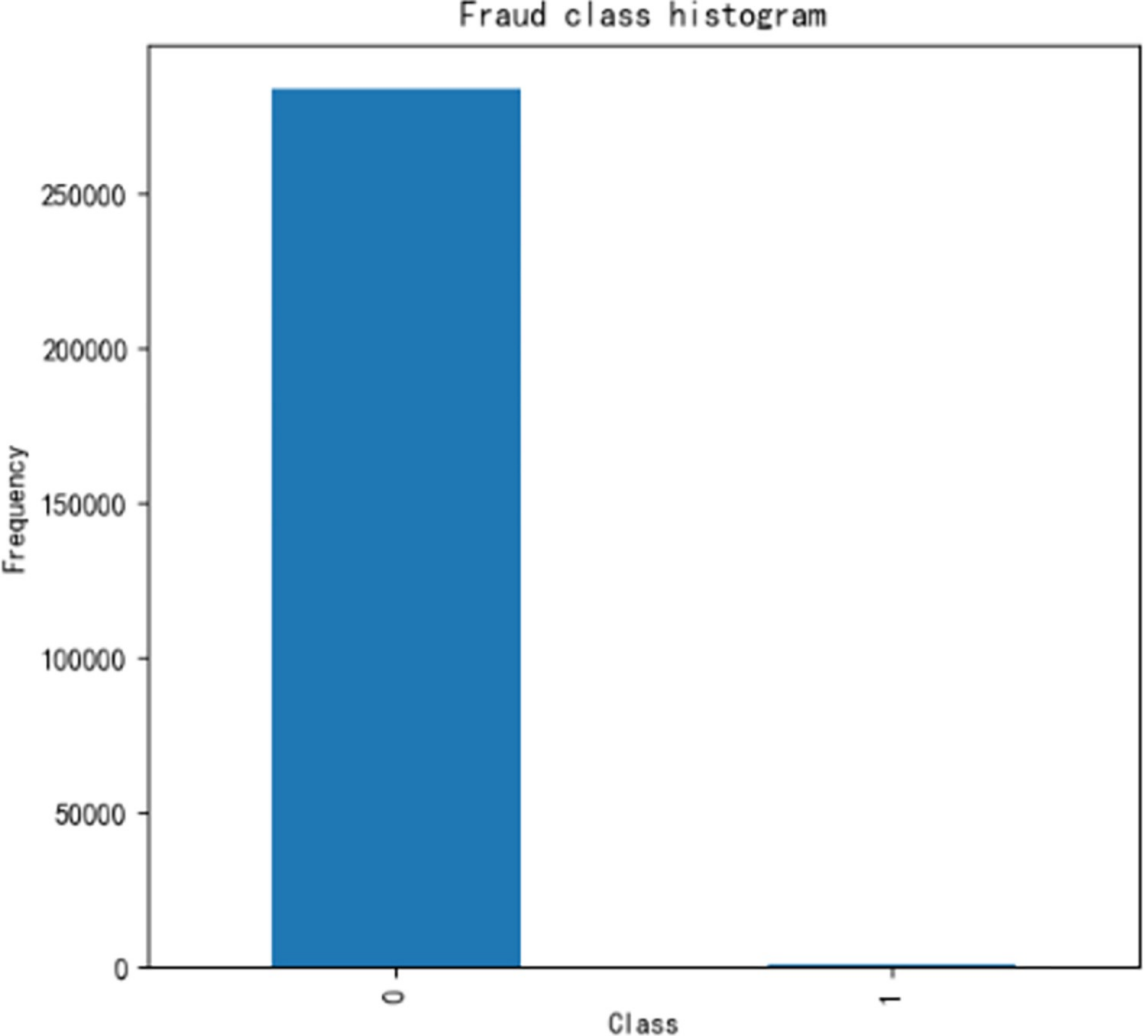
Fraud class histogram before oversampling.

We performed data analysis on the class label histogram. As shown in Fig 6, the data is imbalanced, and if the imbalanced data is directly used for training, it will often lead to inaccurate predictions.

**Fig 7:**
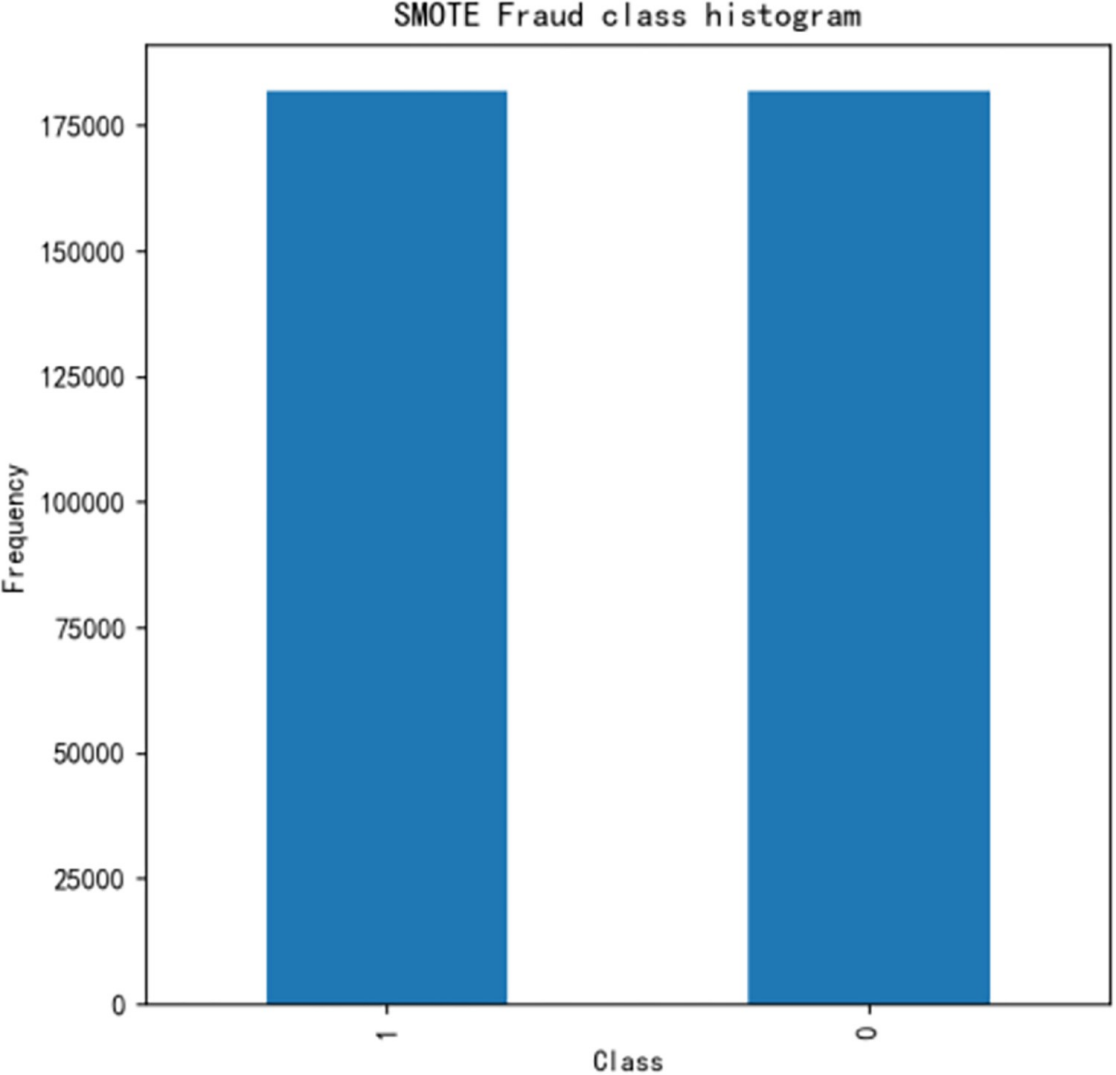
Fraud class histogram after oversampling.

The number of sample data for 0 is much larger than that for 1. To balance the two sampled data, we introduce the smote over-sampling algorithm, which increases the amount of sampled data for class 1, as shown in Fig 7.

After oversampling, the number of class 1 and class 0 is obviously the same. However the smote algorithm has the following shortcomings: firstly, if there are outliers or noise in the samples, the quality of the generated samples may be poor, and new noise may be introduced, which is not conducive to data classification; secondly, the new samples generated by the algorithm may be distributed at the edge of the original data distribution, making it difficult to distinguish between sample boundaries; thirdly, the new samples are only generated between two samples, and the sample generation area is small, which may lead to overfitting. Given the issues with the smote algorithm, the gan algorithm can be introduced. There are several advantages of using GANs over SMOTE algorithm for addressing class imbalance. Firstly, GANs generate synthetic samples by learning the underlying data distribution, which results in more realistic and diverse samples compared to SMOTE’s simple duplication of minority instances. Secondly, GANs can handle complex data distributions and generate samples that better represent the underlying data manifold, while SMOTE is limited to linear interpolation between existing samples. But GANs have a limited capacity to generate samples in large quantities, which may be a disadvantage in applications where a large number of synthetic samples are required. Therefore, we propose in this thesis a combination of smote and gan to enhance the quality of the data.

**Fig 8:**
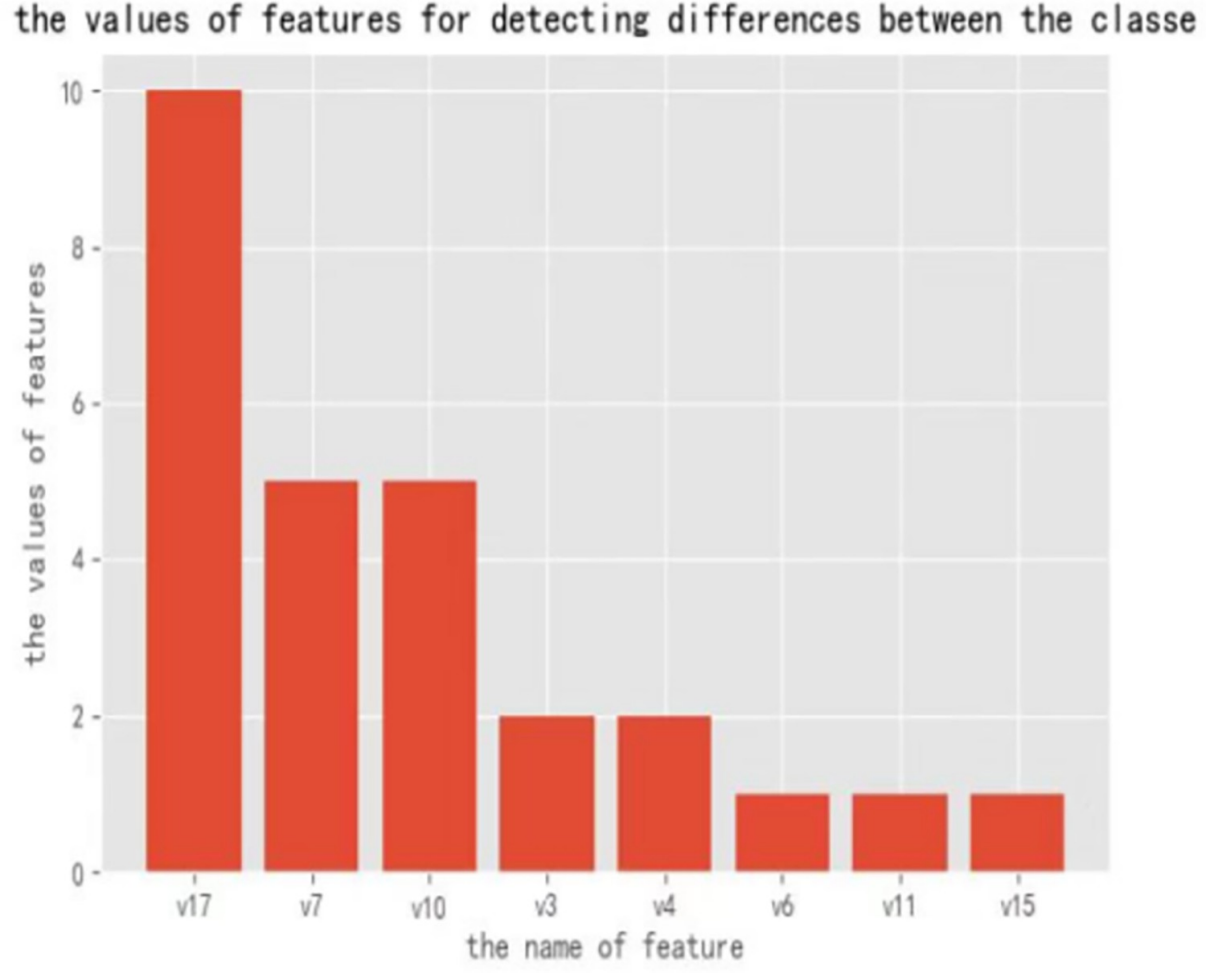
The sorted feature value.

There are more types of gan, and we use cgan here as a means of data enhancement. To indicate that cgan works better than the original gan, we have done a layer of comparative experimentation here. As there are more features in the dataset, we can use certain representative features here to do the comparison experiments. In our experiments, we obtain the feature score of each feature by calling the function get_fscore of the xgboost algorithm [26], and then sort their feature scores from the largest to the lowest k. We get the following display results:

As can be seen from the Fig 8, V11 and V10 have the highest feature values, and we use these two features here as most useful features for detecting differences between the classes

**Fig 9:**
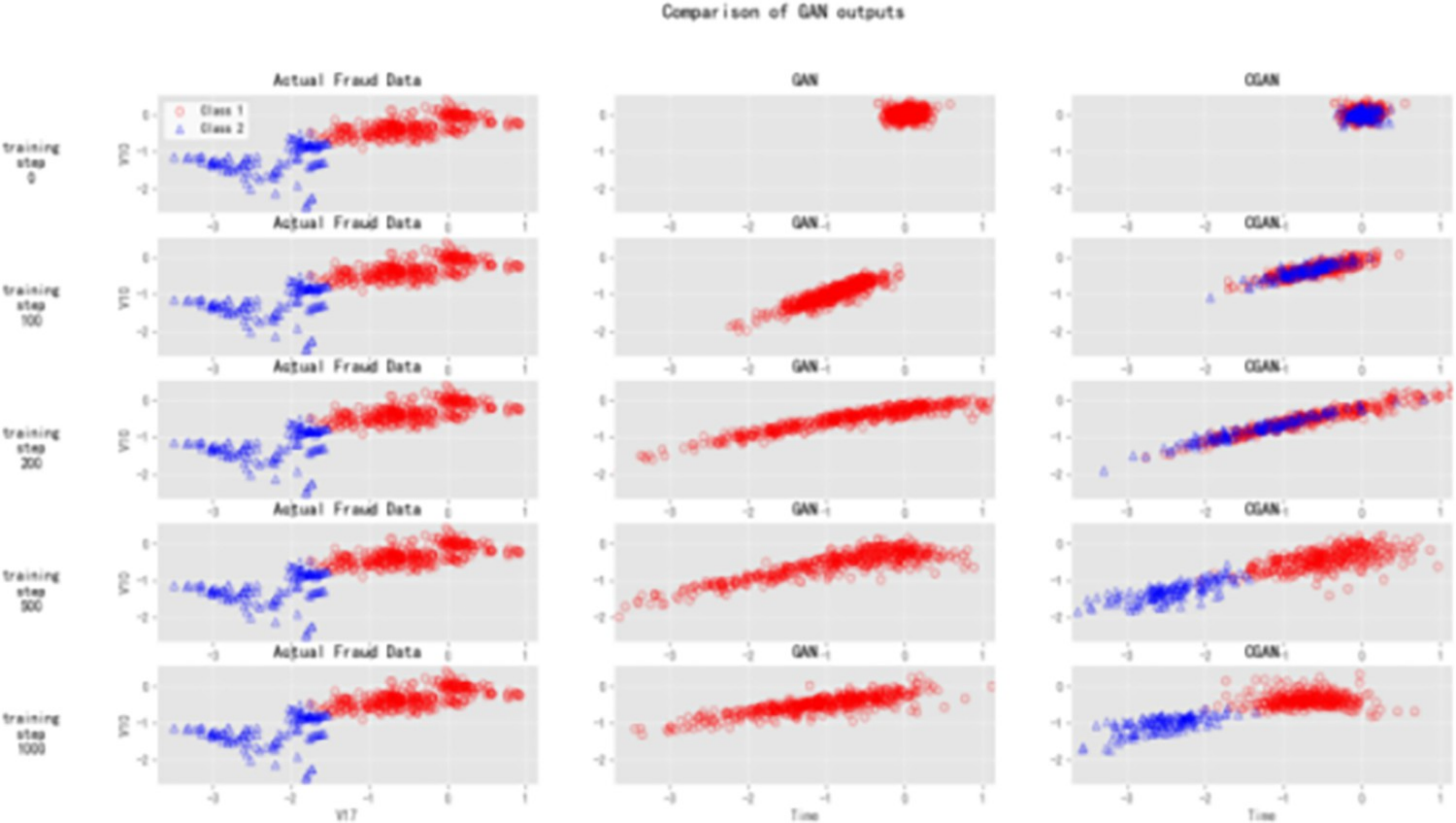
Comparison of GAN outputs.

We put the data generated with the smote algorithm as input data into the gan and cgan networks respectively for training, and then compared them with the original dataset. We train each GAN for 1000 iterations and monitor the progress of the generated fraud data. In Fig 9, we observe the fraud data and the generated fraud data by different GAN architectures during training. Specifically, we divide the actual fraud data into 2 classes using K Means and plot them based on the 2 most significant features (V10 and V17 from PCA transformation). The original GAN ignores class information and generates fraud data all belonging to the same class. However, the conditional CGAN generates fraud data by class.

As the training progresses, we notice that the original GAN architecture starts to recognize the shape and range of the actual data but eventually collapses into a small distribution. This phenomenon is known as mode collapse, where the generator learns a limited range of data that the discriminator has difficulty distinguishing as fake. On the other hand, the CGAN architecture performs slightly better as it spreads out the generated data and approaches the distributions of each class of fraud data.

### 4.2 Performance evaluation of the AE-XGB-SMOTE-CGAN algorithm

The following is the part of designing and verifying the idea of the AE-XGB-SMOTE-CGAN algorithm we proposed. We will describe how to design the AE-XGB-SMOTE-CGAN algorithm in detail and draw conclusions through comparative experiments.

Moving forward, we employed stratified sampling to divide the preprocessed dataset into three subsets: training, validation, and test sets, with a ratio of 6:2:2. We then conducted experiments in accordance with the aforementioned AE-XGB-SMOTE-CGAN algorithm flow.

In terms of the architecture design of the CGAN model, the generator neural network of the CGAN consists of 3 hidden layers, each with 128 neurons. On the other hand, the CGAN discriminator network is similar to the generator network, but with the main difference being that it only has two layers: a linear layer followed by a leaky-ReLu layer with an alpha value of 0.2. The parameter settings, such as the learning rate, total epochs, and loss functions, are presented in Table 2. During CGAN training, a binary cross-entropy activation function is employed, with a training data batch size of 128 and an initial learning rate of 0.0005, utilizing the Adam optimizer.

**Table 2:** Performance of AE-XGB-SMOTE-CGAN with and without data augmentation.

Parameter	Generator	Discriminator
Neural network structure layers	128,256,512,1024	1024,512,256,128
Loss Function	binary_cross_entropy_with_logits	binary_cross_entropy_with_logits
optimizer	Adam	Adam
Activation	RELU	RELU
Learning Rate	0.0005	0.0005

Following the fundamental pre-processing steps, SMOTE oversampling is carried out with 6 neighbors (k = 6). The stopping criteria for training SMOTE-CGAN and naive CGAN models are determined based on the validation error to prevent overfitting. Moreover, it is confirmed that the discriminator and generator loss remain substantial and do not approach zero.

**Fig 10:**
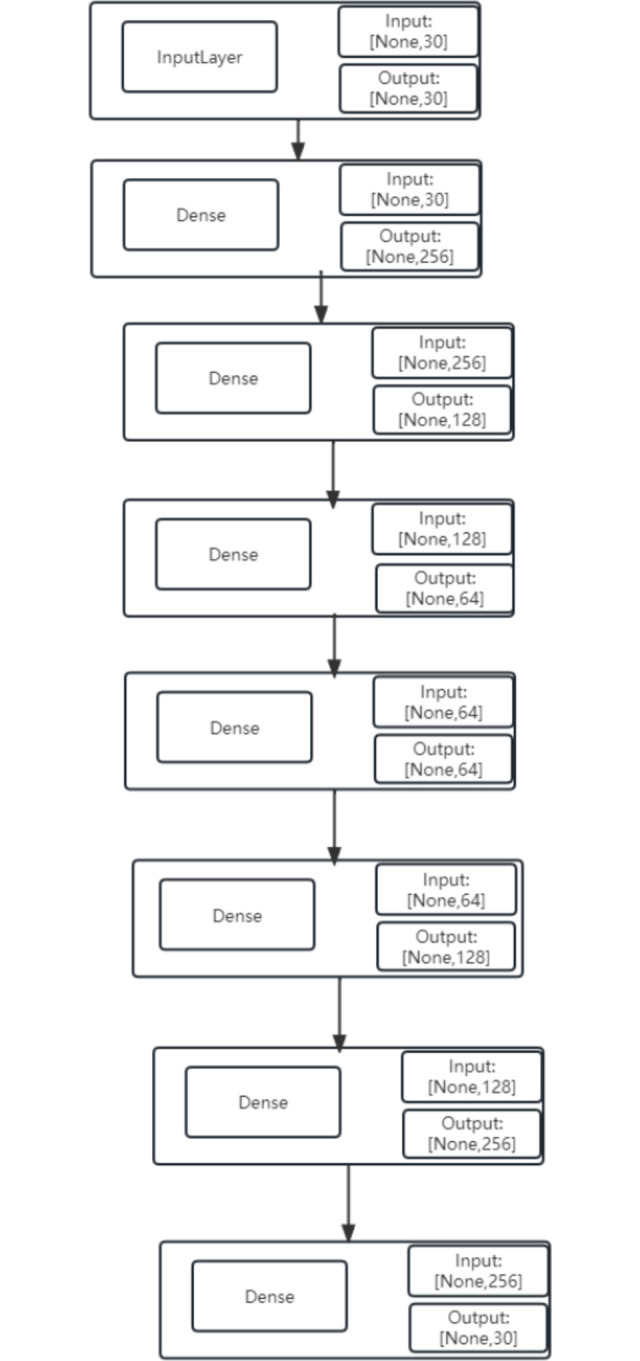
The network architecture design of the autoencoder.

Regarding the architecture design of the autoencoder model, we designed a total of seven neural network layers, as shown in Fig 10. It is crucial to note the dense information displayed in the figure, where the output of the previous neural network layer is connected to the input of the subsequent layer, and the number of neurons in both the input and output are identical. For example, the first dense input has a size of 30, with an output size of 256, while the subsequent layer’s input size is 256, with an output size of 128.

**Fig 11:**
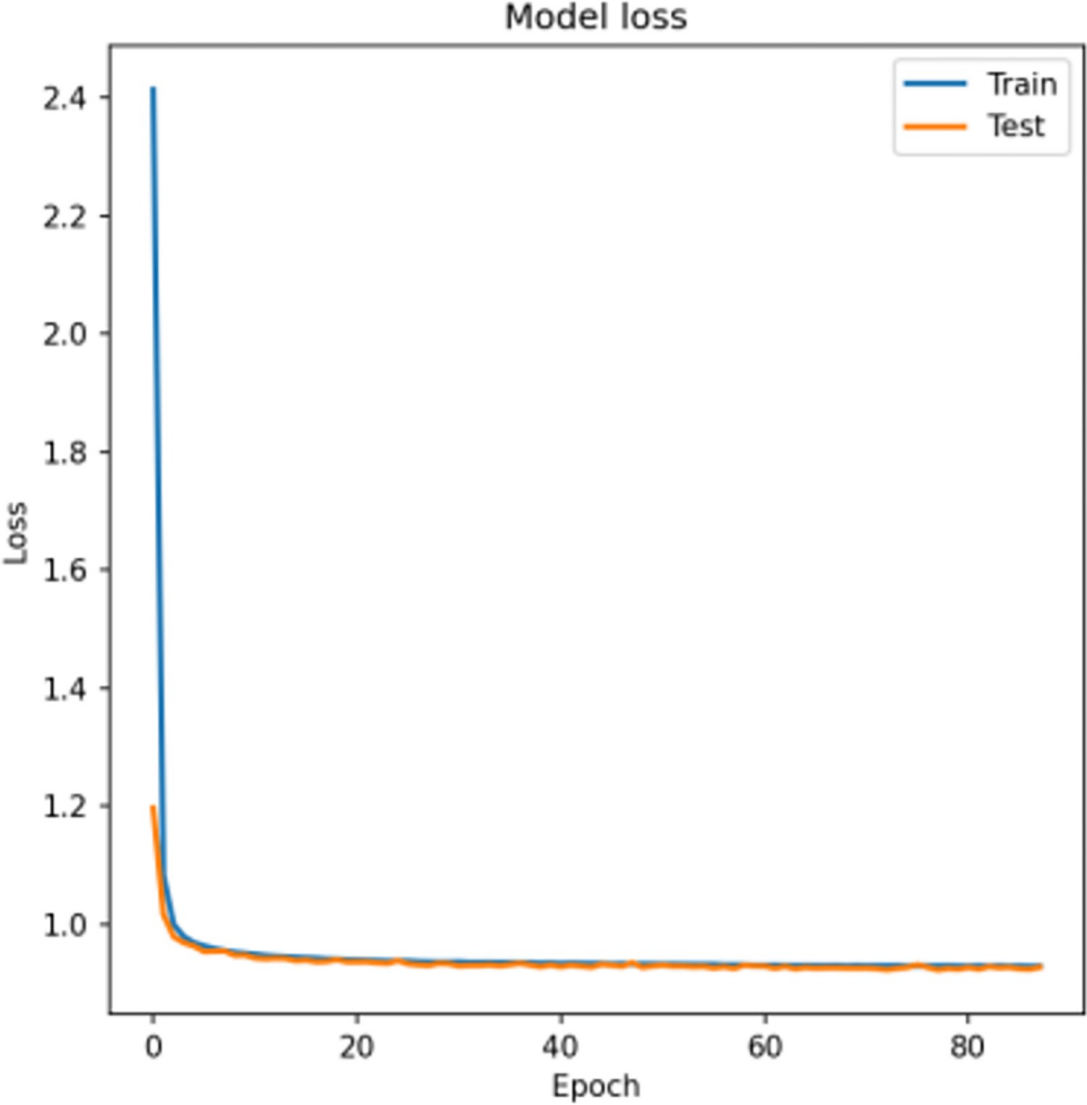
The comparison of the test and training losses.

The activation function used in this study is Mish, a deep learning activation function based on Diganta Misra’s paper "Mish: A Self Regularized Non-Monotonic Neural [27] Activation Function". Compared to ReLU, Mish performs better at high significance levels (p < 0.0001). The autoencoder model is optimized using adam, and L2 regularization is applied to every layer of the encoder and decoder to prevent overfitting. Early stopping is used during training to monitor the validation loss and prevent overfitting. The loss function employed is MSE. For the xgboost model, maximum depth is set to five, learning rate to 0.1, and the number of iterations is seven. It classifies the test data as fraudulent with probability p, where 0 ≤ p ≤ 1, generating probabilistic classification. The performance evaluation of the AE-XGB-SMOTE-CGAN algorithm is conducted in two experiments, one for the data before augmentation and another for the data after augmentation. The comparison of the test and training losses in the training process is shown in Fig 11.

After around 20 training rounds, the training set and test set become very similar, indicating a good fit. XGBoost uses probability classification to determine if test data is fraudulent or not. If the probability (p) is higher than a pre-set threshold value (θ), the data is deemed fraudulent. Different thresholds will result in varying classification performances. To determine the best threshold for specific requirements, fine-tuning and shifting of the threshold is necessary, and the best threshold is identified by specific metrics such as MCC, ACC, TPR, and TNR. The following formulas for these metrics are used to evaluate model performance.


$$TNR=\frac{TN}{TN+FP}$$ (7)

$$FPR=\frac{FP}{TN+FP}$$ (8)

$$TNR=\frac{TN}{TN+FP}$$ (9)

$$TPR=\frac{TP}{TP+FN}$$ (10)

$$ACC=\frac{TP+TN}{TP+TN+FP+FN}$$ (11)

$$MCC=\frac{TP\times TN-FP\times FN}{\sqrt{\left(TP+FP)\times (TP+FN)\times (TN+FP)\times (TN+FN\right)}}$$ (12)

Confusion matrices [28] are often utilized as significant indicators in the field of machine learning to assess the effectiveness and accuracy of a model. In Eqs (7)–(10), TP defines true positive, which represents the accurate prediction of a positive label by the model. FN represents false negative, which indicates the model’s failure to predict a positive label accurately. FP represents False Positive, which means the model has predicted a positive label incorrectly. TN stands for true negative, which indicates the accurate prediction of a negative label by the model. TPR (True Positive Rate), also known as recall, hit rate, or sensitivity, represents the proportion of accurately predicted positive samples. TNR (True Negative Rate), also known as specificity, is the proportion of accurately predicted negative samples. FPR (False Positive Rate) is often used as the x-axis of the ROC curve. Eq (12) represents MCC (Matthew’s correlation coefficient), which is a comprehensive assessment metric that takes into account all four basic confusion matrix indicators. The Matthews Correlation Coefficient (MCC) is a measure of the correlation between the predicted and actual samples. Its value ranges from -1 to 1, where a value of 1 indicates perfect predictions, 0 indicates worse than random predictions, and -1 indicates almost perfect avoidance of the correct answer. MCC is a comprehensive metric and is considered the best for binary classification problems [29]. TPR is also an important metric, especially for fraud detection, as a higher TPR means more fraud data can be detected. However, when evaluating the overall performance of the model, MCC is considered as it takes into account all parameters such as TP, TN, FP, and FN.

**Fig 12:**
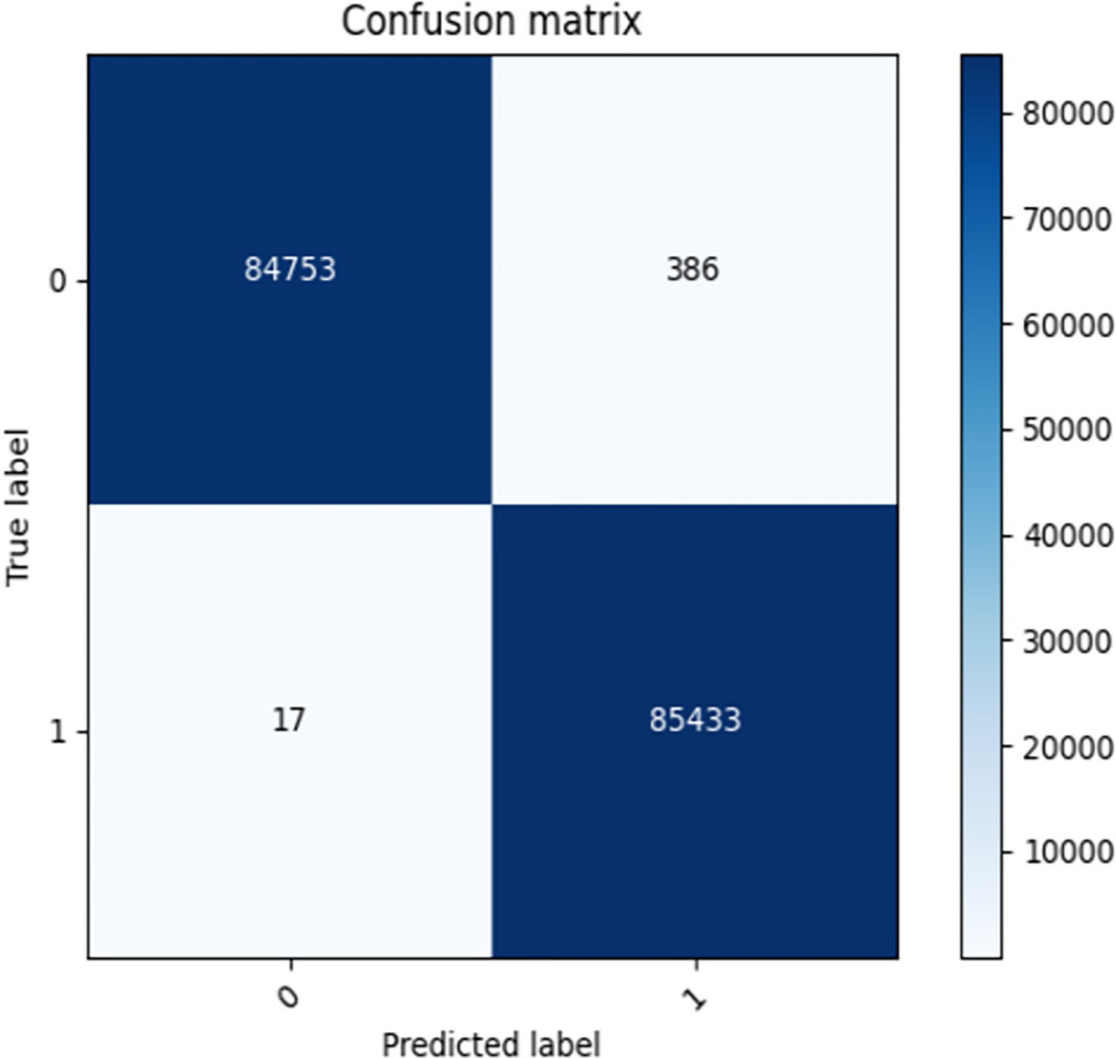
The confusion matrix for AE-XGB-SMOTE-CGAN method.

Fig 12 shows the graph of confusion matrix, From the graph, it can be seen that the number of TP and TN is large, while the number of FP and FN is small, indicating that the model’s predictive performance is relatively good.

**Fig 13:**
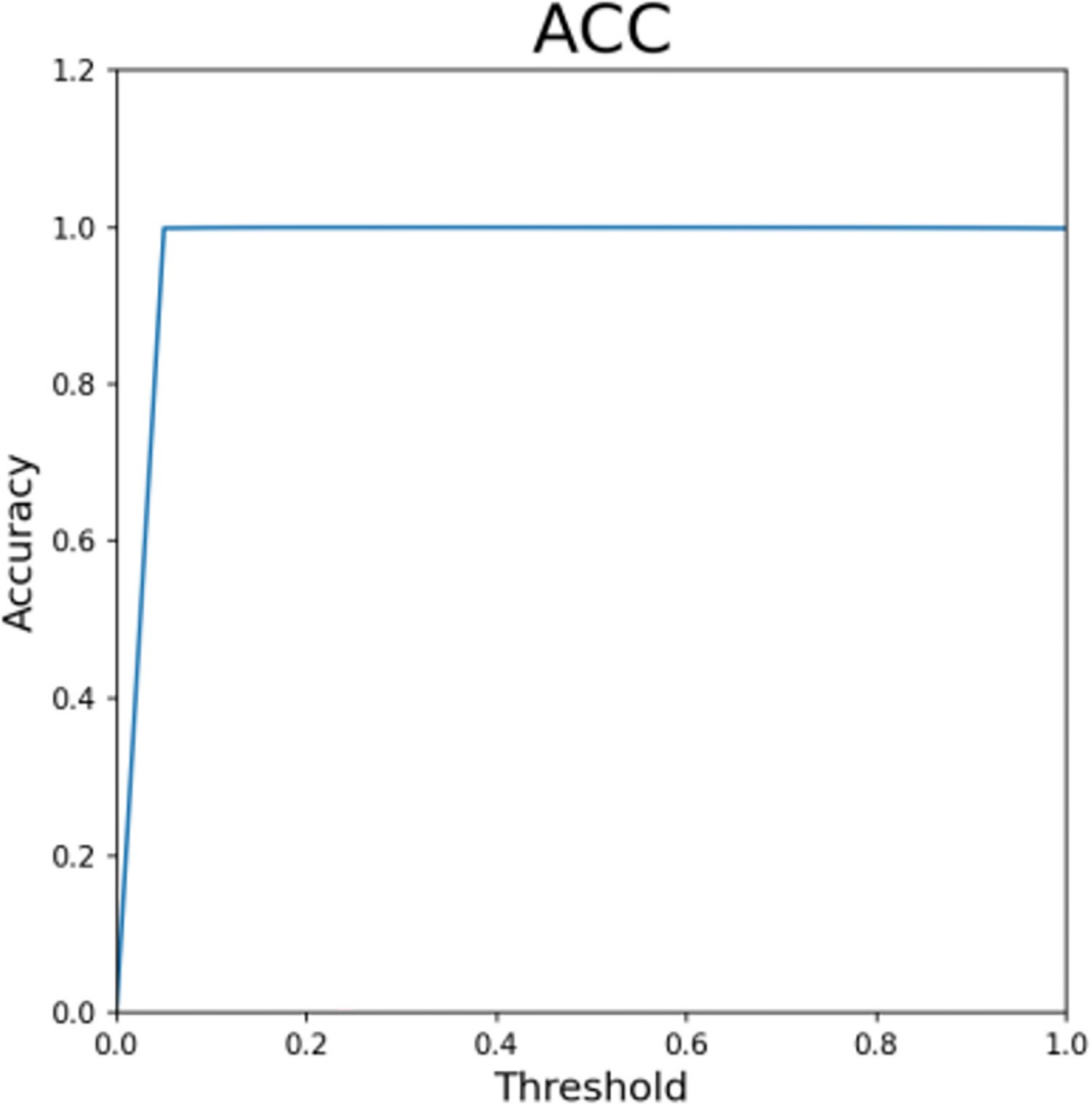
The ACC for different thresholds.

**Fig 14:**
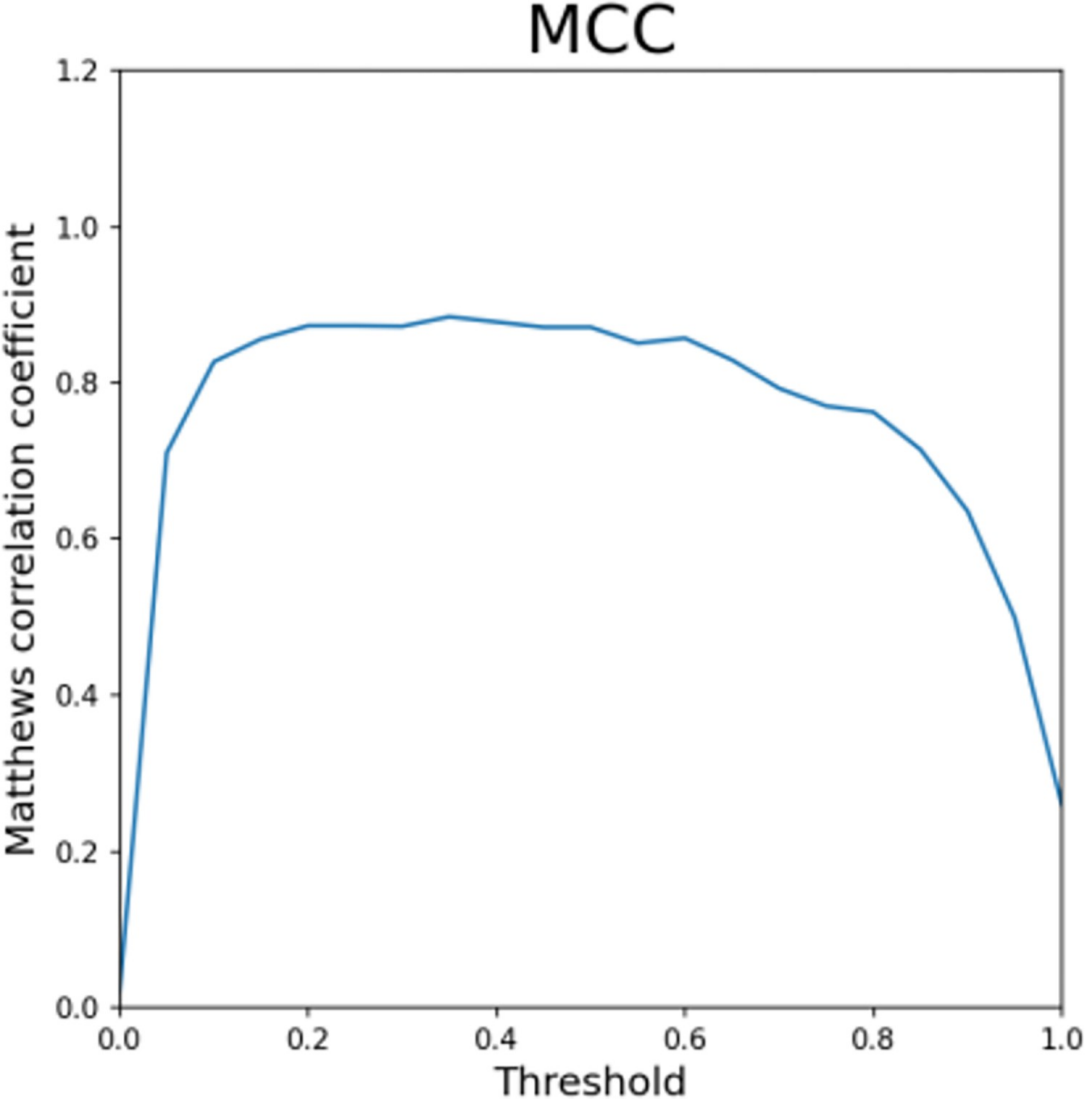
The MCC for different thresholds.

**Fig 15:**
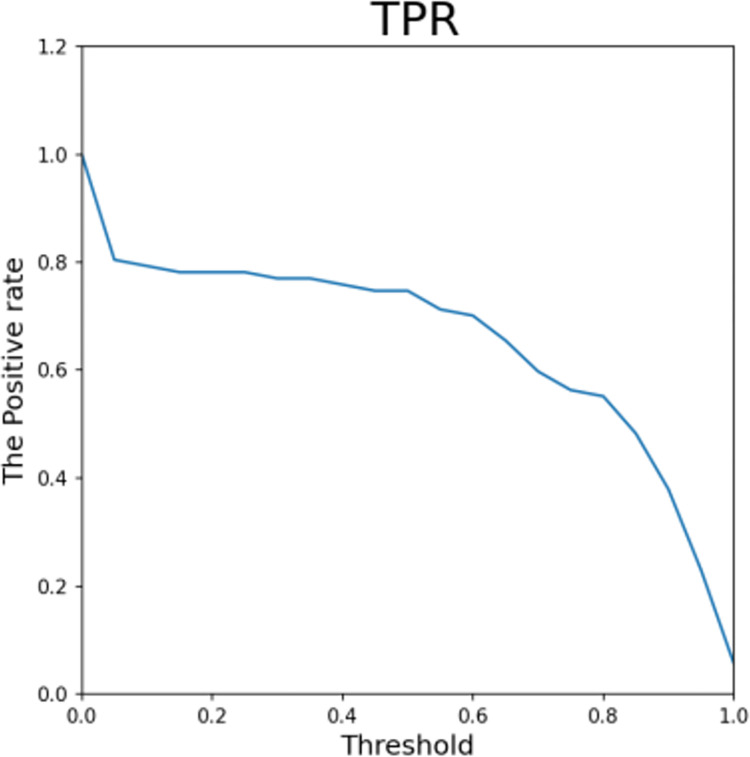
The TPR for different thresholds.

**Fig 16:**
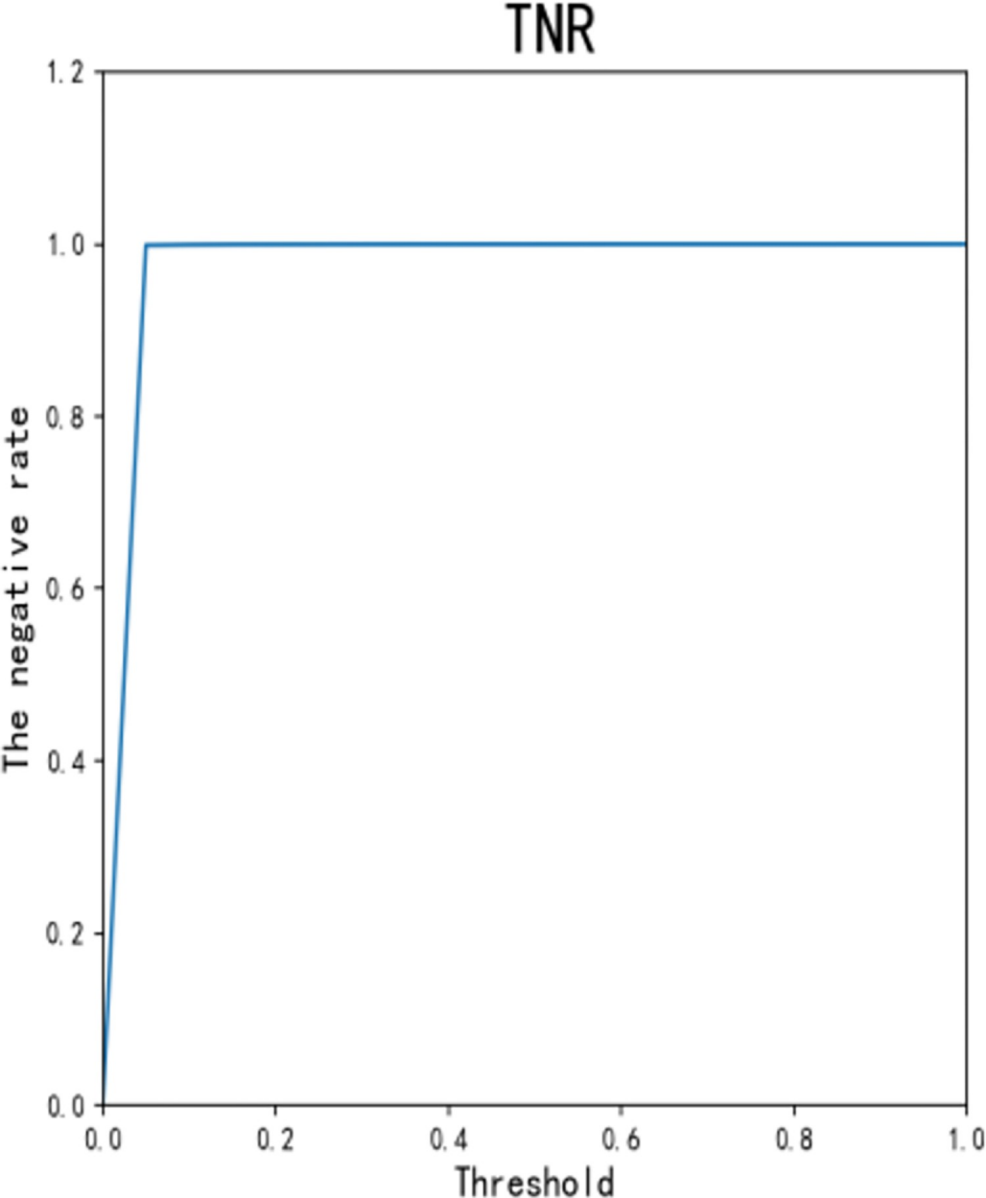
The TNR for different thresholds.

Figs 13–16 shows the curve graphs of ACC, MCC, TPR [30], and TNR at different threshold values. The AE-XGB-SMOTE-CGAN classifier was evaluated using 101 different threshold values ranging from 0 to 1 by incrementing 0.01. ACC values were consistently high at above 0.9995, except for a value of 0.0017 when the threshold was 0. TNR values were also consistently high at 0.999 except when the threshold was 0. From the MCC curve, it was observed that a threshold between 0.2 to 0.8 yielded an MCC value greater than 0.8. The maximum MCC value of 0.8845 was obtained at a threshold around 0.35. However, in credit card anti-fraud problems, TPR, which represents the proportion of correctly predicted fraudulent data, is also an important metric. Considering both MCC and TPR, a threshold of 0.2 was found to be most appropriate as both metrics exceeded 0.8. A threshold of 0.05 could also be considered as it yielded an MCC value greater than 0.5 and a TPR value close to 0.9.

For the second stage of the experiment, we will solely utilize the smote algorithm to balance the original data by resampling it. We will then repeat the first stage of the experiment to compare the indicator values between the resampled data set and the original data set without resampling, along with the data set enhanced by both smote and cgan algorithm. If the indicator values of the smote-cgan algorithm are higher than those of the other data sets, then it will suggest that the AE-XGB-SMOTE-CGAN algorithm is better suited for processing unbalanced data sets. After conducting 100 experiments and averaging the four indicators, we obtained comparative Table 3 by comparing the experimental data before and after data resampling and data augmentation.

**Table 3:** Performance of AE-XGB-SMOTE-CGAN with and without data augmentation.

Models	ACC	TPR	TNR	MCC
AE-XGB-SMOTE-CGAN (threshold = 0.35)	0.9993	0.7839	0.9997	0.8845
AE-XGB-SMOTE-CGAN(threshold = 0.05)	0.9987	0.8929	0.993	0.773
AE-XGB-SMOTE (threshold = 0.70)	0.9970	0.8275	0.9973	0.5574
AE-XGB(threshold = 0.2)	0.9975	0.8456	0.9993	0.8506

The AE-XGB-SMOTE method performs best when the threshold is set to 0.70, but its MCC score falls short compared to that of AE-XGB-SMOTE-CGAN. Additionally, there are no significant improvements in terms of ACC and TPR. Based on these comparison experiments, it can be inferred that the AE-XGB-SMOTE-CGAN approach is better suited for addressing imbalanced datasets.

To further validate the superior performance of the AE-XGB-SMOTE-CGAN algorithm, additional experiments were conducted to compare it with other methods. The commonly used XGBoost, KNN, and Random Forest algorithms were selected for these experiments, and the dataset was again used prior to sampling. Performance indicators including ACC, TPR, TNR, and MCC were utilized. The comparison results are presented in Table 4.

**Table 4:** Performance comparisons of AE-XGB-SMOTE-CGAN and related methods.

Models	ACC	TPR	TNR	MCC
AE-XGB-SMOTE-CGAN (threshold = 0.35)	0.9993	0.7839	0.9997	0.8845
AE-XGB-SMOTE-CGAN (threshold = 0.05)	0.9987	0.8929	0.9932	0.773
KNN	0.9691	0.8835	0.9711	0.5903
XGBoost	0.9696	0.8529	0.9995	0.8100
Random Forest	0.9583	0.8025	0.9989	0.6902
AE-based clustering	0.98902	0.81632	0.98932	0.3058

Based on the results presented in the table, it can be inferred that AE-XGB-SMOTE-CGAN exhibits superior performance compared to the other methods. This is evident from its high MCC, ACC, and TNR values, particularly when using a threshold of 0.35. However, its TPR values are lower than KNN and xgboost algorithms. Although the highest TPR value for AE-XGB-SMOTE-CGAN is at a threshold of 0.05 (0.8929), its MCC value is comparatively lower than AE-XGB (threshold = 0.2). If credit card fraud detection primarily values TPR, then AE-XGB-SMOTE-CGAN (threshold = 0.05) is the recommended option. Additionally, at 0.773, its MCC value is higher than 0.5 and outperforms the KNN, XGBoost, AE-based clustering [31] and Random Forest models.

### 4.3 Discussion and application

Based on our experiments, we have determined that the AE-XGB-SMOTE-CGAN algorithm is more adept at handling imbalanced datasets and yields superior results when compared to conventional machine learning techniques, like KNN, XGBoost, and random forest. Notably, the AE-XGB-SMOTE-CGAN algorithm improves the ACC index by 2% compared to xgboost and KNN, and when the threshold is set at 0.35, the MCC index of AE-XGB-SMOTE-CGAN is 8% higher than the second-highest performing xgboost algorithm, and 30% higher than KNN’s.

Our comparison with the xgboost model demonstrated that adding the autoencoder enhances feature learning abilities. However, there are still areas where the AE-XGB-SMOTE-CGAN algorithm can improve, such as the model’s training complexity and MCC and TPR values. We aim to enhance the AE-XGB-SMOTE-CGAN performance further by fine-tuning the hyperparameters of the autoencoder and xgboost models. Currently, the algorithm has been implemented in credit card fraud detection at a bank; we aim to utilize it in more risk control scenarios in the future.

## 5. Conclusions

The purpose of this research paper was to propose an AE-XGB-SMOTE-CGAN algorithm to address the problem of bank credit card fraud. The proposed algorithm utilized an autoencoder to extract feature data and then applied the xgboost algorithm for classification and prediction. During the training of the model, the optimal threshold value was obtained by traversing different threshold values and comparing various indicator parameters. Any data with a value exceeding the threshold was identified as fraudulent. The evaluation of the AE-XGB-SMOTE-CGAN algorithm was performed using an anonymized dataset from a bank with imbalanced class distribution. However, the dataset is imbalanced, with a significantly larger number of normal data instances with class 0 compared to fraudulent data instances with class 1. To solve this issue, the SMOTE algorithm was utilized for oversampling, but its quality was limited. To improve the quality of the generated samples, the CGAN algorithm was introduced. However, CGAN is not the most suitable option for oversampling, as it was originally developed to generate realistic images using convolutional neural networks (CNNs), rather than producing oversamples for minority classes. We have introduced a data augmentation strategy that integrates GAN and SMOTE features to tackle class imbalance in pattern classification problems. Based on the experimental results, the AE-XGB-SMOTE-CGAN algorithm outperformed AE-XGB-SMOTE and AE-XGB algorithms, indicating its suitability for handling unbalanced data in bank fraud. We also compared our algorithm with KNN, Random Forest, and XGBoost algorithms, it showed that the AE-XGB-SMOTE-CGAN algorithm had the highest MCC, TNR, and ACC values at a threshold of 0.35 and the highest TPR value at a threshold of 0.05. Therefore, we recommend setting the threshold at 0.05 to identify more fraudulent data. In future studies, we plan to apply our algorithm to other bank risk control datasets to further optimize its performance and evaluate its generalizability. It will be also interesting to investigate the conjoining of GAN with other over-sampling techniques such as MCMC [32] and WGAN,WCGAN,DRAGAN and so on.
